# Phytochemical, antioxidant and antibacterial properties of *Melissa officinalis* and *Dracocephalum moldavica* essential oils

**Published:** 2017-09-15

**Authors:** Ali Ehsani, Omar Alizadeh, Mohammad Hashemi, Asma Afshari, Majid Aminzare

**Affiliations:** 1 Department of Food Science and Technology, Faculty of Nutrition, Tabriz University of Medical Sciences, Tabriz, Iran;; 2 Department of Food Hygiene and Quality Control, Faculty of Veterinary Medicine, Urmia University, Urmia, Iran;; 3 Department of Nutrition, Faculty of Medicine, Mashhad University of Medical Sciences, Mashhad, Iran;; 4 Department of Food Safety and Hygiene, School of Public Health, Zanjan University of Medical Sciences, Zanjan, Iran

**Keywords:** β-carotene bleaching tests, *Deracocephalum moldavica*, *Melissa officinalis*, Micro dilution

## Abstract

Aromatic plants are rich in essential oils with considerable antimicrobial properties. The aim of this study was to investigate chemical composition, antimicrobial activity and antioxidant properties of *Melissa officinalis* and *Deracocephalum moldavica *essential oils (EOs). The identification of chemical constituents of the EOs was carried out using gas chromato-graphy-mass spectrometry analysis and antimicrobial activity of the EOs was evaluated by disc diffusion assay as well as determination of minimal inhibitory concentration (MIC) and minimal bactericidal concentration against four important food-borne bacteria: *Salmonella typhimorium, Escherichia coli, Listeria monocytogenes *and* Staphylococcus aureus*. Antioxidant activity of the EOs was also determined by 2,2-diphenyl-1-picrylhydrazyl, 2,2-azinobis 3-ethylbenzo thiazoline-6-sulfonic acid and β-carotene bleaching tests. The major compounds of *D. moldavica *were geranial (28.52%), neral (21.21%), geraniol (19.60%), geranyl acetate (16.72%) and the major compounds of *M. officinalis* EO were citronellal (37.33%), thymol (11.96%), citral (10.10%) and β-caryophyllene (7.27%). The underlying results indicated strong antimicrobial effects of the oils against tested bacteria. *Staphylococcus aureus* with the lowest MIC value (0.12 mg mL^-1^) for both EOs was the most sensitive bacterium, although, antibacterial effect of *M. officinalis* EO was stronger than *D. moldavica*. In addition, the results of the antioxidant activity showed that both EOs had notable antioxidant properties. In conclusion, both EOs are appropriate alternatives as potential sources of natural preservative agents with the aim of being applied in food industries.

## Introduction

Concerns regarding the safety of chemical preservatives and the negative reactions of consumers against these components have led to an increasing interest in the use of natural preservatives, such as plant EOs and probiotics.^1^^,^^2^ Some of the most considerable aspects of the functions of plant EOs are the pathogen growth inhibition, delaying food spoilage and the improvement of organoleptic quality. Using natural products as antibacterial compounds can reduce health hazards and economic losses that have been produced by foodborne microorganisms.^3^ The EOs and their components are known to be active against a wide variety of microorganisms, including gram-negative^4^ and gram-positive bacteria.^5^ Gram-negative bacteria are shown to be generally more resistant than gram-positive ones to the antagonistic effects of EOs because of the presence of lipopolysaccharide in their outer membrane.^6^

Among these products, lemon balm (*Melissa officinalis*, Lamiaceae) is a medicinal plant and used for the treatment of headaches, gastrointestinal disorders, nervousness, and rheumatism. It is an antibacterial and antifungal agent.^7^ Previous studies exhibited synergistic effects of *Melissa officinalis* with some preservatives like sodium benzoate, potassium sorbate and sodium nitrite.^8^ *Moldavian dragonhead* (*Dracocephalum moldavica*) is also consumed commonly as a food-related product with medicinal properties; it is effective against liver and stomach disorders, headaches and congestion.^9^

The objectives of this study were (1) to study the chemical constitution of hydro-distilled EOs of *M. officinalis* and *D. moldavica *(2) to investigate the antimicrobial activity of *M. officinalis* and *D. moldavica *EOs by disc diffusion and broth micro-dilution assays against *Escherichia coli*, *Salmonella typhimurium*, *Staphylococcus aureus* and *Listeria monocytogenes *(3) to evaluate antioxidant capacity of *M. officinalis* and *D. moldavica *EOs by 2,2-diphenyl-1-picrylhydrazyl (DPPH), 2,2-Azinobis 3-ethylbenzo thiazoline-6-sulfonic acid (ABTS) and β-carotene bleaching tests and to find effective natural agents for protection against oxidation changes and pathogenic bacteria of food.

## Materials and Methods

**Plant material and EOs preparation. **Aerial parts of *M. officinalis* and *D. moldavica* plants were collected (about 10 kg for each plant) at blossoming stage in the summer 2013 in Urmia, Urmia, Iran and were confirmed by the Herbarium Department of Jahad Agriculture and Natural Resources Center of West Azerbaijan, Iran. The extraction of the EOs were performed using the hydro-distillation method.^10^ Briefly, 100 g of the dried parts of *M. officinalis* and *D. moldavica* plants were separately grounded and placed into water (900 mL) in distillation flasks. Each flask was coupled to a Clevenger type apparatus and heated at 100 ˚C for three hr and finally, the upper liquid (i.e. EO) was isolated from the Clevenger apparatus. This procedure was repeated several times to obtain sufficient EOs for further experiments. The obtained EOs were dehydrated over anhydrous sodium sulfate, filtered by 0.22 μm filters and stored at 4˚C. The yield of EOs were calculated by weighting the obtained EOs each time and then reported as percentage of EOs per 100 g of plants.^10^

**Gas chromatography-mass spectrometry (GC-MS) analysis. **The GC/MS analysis of EOs were performed using a Hewlett Packard 5890 (Hewlett Packard Corporation, Palo Alto, USA) equipped with an HP-5MS capillary column (30 × 0.25 mm inner diameter and 0.25 mm film thickness). The Helium flow rate was one mL per min. The column temperature was initially 50 ˚C and then gradually increased to 120 ˚C at 2 ˚C per min rate, held for 3 min, and finally increased to 300 ˚C. The MS procedure was operated through ionization energy of 70 eV. Thereafter, the compounds were identified by comparing their retention indices with those of authentic samples and the mass spectral data available in the library (Wiley-VCH 2001 data software, Weinheim, Germany).^11^

**Evaluation of antibacterial activity. **Antibacterial activity of the EOs was investigated against four important food-borne bacteria including *E. coli* (PTCC 1533), *L. monocytogenes *(PTCC 1298), *S. aureus *(PTCC 1015) and *S. typhimurium *(PTCC 1730). The bacterial strains were obtained from a microbial collection of Department of Food Hygiene and Quality Control, Faculty of Veterinary Medicine, Urmia University, Urmia, Iran. Bacterial suspensions were prepared to culture the lyophilized bacteria in 9 mL of Brain heart infusion (BHI) broth (Sigma-Aldrich, St. Louis*, *USA) and incubated at 37 ˚C for 24 hr.^12^

**Agar disk diffusion assay. **Sterile paper disks (6 mm in diameter) were impregnated with 20 μL of two concentrations (10 and 15 mg mL^-1^) of *M. officinalis *and *D. moldavica *EOs and then placed on the surface of the nutrient agar plates inoculated with 0.1 mL of the bacterial cultures (1.5 × 10^6 ^CFU mL^-1^) under aseptic condition. The plates were then incubated at 37 ˚C for 24 hr and the diameters of inhibition zones were measured using a caliper. Vancomycin and ampicillin antibiotic disks were used as control positives.^12^

**Micro-well dilution assay. **Micro-well dilution assay was used to determine minimal inhibitory concentration (MIC) and minimal bactericidal concentration (MBC) of the EOs against the tested bacterial strains (*E. coli*, *L. monocytogenes*, *S. aureus *and *S. typhimurium*). Bacterial suspensions were prepared while being in log phase, conformed to the 0.5 McFarland standard turbidity and serially diluted (1:10) to achieve the desired concentration (1.5 × 10^6 ^CFU mL^-1^). The EOs were dissolved in 10% dimethyl sulfoxide, then the solutions were prepared at the concentration of 40 mg mL^-1^ as a stock solution, and then the serial two-fold dilutions were made in a concentration range from 0.62 to 40 mg mL^-1^in nutrient broth. Briefly, 160 μL of the nutrient broth, 20 μL of the inoculums and 20 μL of the EOs were added into each well.^13^ Subsequently, the wells without any bacteria and the wells without any EOs were considered as negative and positive controls, respectively. The microplates were mixed gently and incubated at 37 ˚C for 24 hr. The final volume in each well was 200 µL, the final concentrations of EOs were in a range between 0.062 to 4.000 mg mL^-1^ and the final bacterial suspensions in the wells were approximately 1.5×10^5^ CFU mL^-1^. The lowest concentration with no visible bacterial growth was regarded as MIC values of the EOs. The MBC values were determined by inoculating 10 μL of none-turbid wells on BHI agar, while the lowest concentration with no visible bacterial growth on the agar was *regarded* as MBC values of the EOs.^12^^,^^14^

**Determination of antioxidant activity. **The possible antioxidant activity of the EOs was assessed by three basically different systems: DPPH radical scavenging activity (RSA) assay, β-carotene bleaching test (BCBT) and ABTS assay.

The DPPH assay was performed by adding 2 mL of methanolic DPPH solution (24 µg mL^-1^) to 50 μL of various concentrations of the EOs (1.0, 2.5, 5.0 and 10 mg mL^-1^) in methanol. The absorbance of prepared solutions and the blank, containing the same chemicals without any antioxidant, were recorded at 517 nm (Pharmacia LKB Novaspec, Uppsala, Sweden), after an incubation period of 60 min at room temperature in a dark place. Moreover, the *capacity* of the EOs to scavenge DPPH radicals was calculated as follows: ^15^

*RSA (%) =[( A *_blank_ *– A *_sample_ *)/ A *_blank_ *] × 100*

The BCBT was carried out as previously described with minor modification.^16^ A solution of 0.5 mg β-carotene (type I synthetic; Sigma-Aldrich, St. Louis, USA) in 1 mL chloroform was prepared in a flask. Then 25 µL linoleic acid (Sigma-Aldrich) and 400 mg Tween 40 (Sigma-Aldrich) were added into the flask. The chloroform was removed completely using a rotary evaporator (Model 4003; Heidolph Laborata, Schwabach, Germany) at 40 ˚C and then 100 mL of distilled water was added and shaken vigorously. Aliquots 2.5 mL of this emulsion were transferred into a series of test tubes containing 350 μL of various concentrations (1.0, 2.5, 5.0 and 10 mg mL^-1^) of the EOs. The same procedure was repeated with butylated hydroxytoluene (BHT) and ascorbic acid as reference antioxidants along with a blank. The absorbance of each tube was measured at 470 nm immediately at zero time and also after a two-hour period while the tubes were kept in a water bath at 50˚C. The capacity of the EOs to protect against oxidation of β-carotene was determined as follows:

*Inhibition (%) = A *_β-carotene after 2 hr assay_ */ A *_initial β-Carotene_

Radical scavenging activity of the EOs against ABTS^+^ was measured according to the previously described procedure.^17^ The ABTS solution (7.0 mmol L^-1^) and potassium persulphate solution (2.45 mmol L^-1^) in distilled water were separately prepared and reacted together to produce ABTS radicals. The mixture was kept in the dark at room temperature for 16 hr. The ABTS^+^ solution was diluted with phosphate buffer saline (PBS) to an absorbance of 0.70 at 734 nm. Aliquots of 200 μL of various concentrations (1.0, 2.5, 5.0 and 10 10 mg mL^-1^) of the EOs in methanol and the reference antioxidants (ascorbic acid and BHT) were added to 2 mL of ABTS^+^ solution and mixed vigorously. After an incubation period of 6 min at room temperature, the absorbance was measured at 734 nm and the ABTS^+^ scavenging effect was calculated formulas follows:

*RSA (%) =1*– * (A *_sample_* / A *_blank_*) × 100*

**Statistical analysis. **Statistical analysis of data was performed using SPSS (version 18.0; SPSS Inc., Chicago, USA). Paired t-test was used to compare the differences of mean values among the EOs (*p *< 0.05). All the experiments were carried out in triplicate.

## Results

**Chemical composition. **The yield of EOs for each plant (*M. officinalis* and *D. moldavica*) was about 1.00% (EO mL per g of dry plant). The GC-MS analysis of *M. officinalis* and *D. moldavica* EOs identified 37 (Table 1) and 22 components (Table 2) representing 85.12% and 93.26% of the total contents of the EOs, respectively. The main components of *D. moldavica* EO were geranial (28.52%), neral (21.21%), geraniol (19.60%), geranyl acetate (16.72%), and the major compounds of *M. officinalis* EO were citronellal (37.33%), thymol (11.96%), citral (10.10%) and β-caryophyllene (7.27%).

**Antibacterial activity. **The results of *in vitro* antibacterial activity of *M. officinalis* and *D. moldavica* EOs against tested food borne bacteria strains were assessed by agar disk diffusion and broth micro-well dilution assays, (Table 3). The examined gram negative bacteria (*E. coli* and S. *typhimurium*) were more resistant to the antibacterial activity of the EOs than gram positive bacteria (*S. aureus* and *L. monocytogenes*). Among the tested bacterial strains by disc diffusion assay, *E. coli* and *S. aureus* showed the lowest and the highest sensitivity to the tested concentrations of the EOs, respectively.

**Table 1:** Chemical compositions of *M. officinalis* by gas chromatography-mass spectrometry analysis

**Components**	**Retention time**	**(%)**
**Aromadendrene**	47.12	0.29
**Bicyclo(3.1.1)Heptane**	19.42	0.11
**Bicyclo(2.2.1)Heptane-2-ol**	24.53	0.39
**Bicyclo(2.2.1)Hepten- 2-one**	17.96	0.06
**β-Bisabolene**	33.93	0.15
**Carvacrol**	25.30	0.97
**Β-Caryophyllene**	30.27	7.27
**Citral**	23.97	10.10
**Citronellyl Butyrate**	27.55	0.10
**Citronellal**	18.67	37.33
**Citronellol**	22.01	0.29
**Citronella**	18.80	0.23
**p-Cymene**	12.45	0.13
**2.6- Dimethyle-5-Heptanal**	13.85	0.15
**Eucalyptole**	12.73	0.11
**Germacerene-D**	32.78	0.26
**5-Hepten-1-ol**	17.40	0.13
**α-Humulene**	31.63	0.42
**Isopulegol**	18.05	0.54
**Isoborneol**	19.04	0.21
**β-Ionone**	33.01	0.09
**Linalool**	14.03	3.05
**Limonene**	12.63	0.06
**3-Methyl-2-(2-Methyl-2-Butenyl**	15.87	0.10
**1,3,8-p- Menthatriene**	22.58	7.22
**Myrcene**	11.02	0.07
**α-Muurolene**	36.51	0.70
**Neophytadiene**	46.14	0.27
**1-Octen-3-0l (1-)**	10.52	0.40
**1.3.6-Octatriene**	13.60	0.15
**3.6-Octadienoic acid**	24.16	0.16
**2.6-Octadienoic acid**	26.24	0.61
**Rose oxide**	16.49	0.18
**β-thujone**	16.20	0.27
**γ –Terpinene**	14.01	0.25
**Thymol**	24.96	11.96
**Trans-chrysanthemal**	18.27	0.34
**Total content**	-	85.12

**Table 2:** Chemical compositions of *D. moldavica* determined by gas chromatography-mass spectrometry analysis

**Components**	**Retention time**	**(%)**
**Benzene acetaldehyde**	18.01	0.20
**Bergamal**	18.14	0.07
**Caryophyllene oxide**	42.30	0.24
**Caryophyllene(E)**	35.47	0.13
**α-Copaene**	33.50	0.10
**delta-Cadinene**	39.55	0.06
**Ethyl nerolate**	32.52	0.11
**Geraniol**	28.02	19.60
**Geranial**	29.00	28.52
**Geranyl acetate**	33.67	16.72
**Germacrene-D**	38.08	0.25
**Hepten-2-one(6-methyl-5)**	14.61	0.77
**Linalool**	20.49	0.82
**Linalool oxide**	18.95	0.08
**Methyl geranate**	31.18	0.17
**Myrcene**	3.26	0.10
**Neral**	27.55	21.21
**Nerol**	26.71	1.86
**Neryl acetate**	32.76	1.76
**1-Octen-3-0l (1-)**	14.31	0.25
**Piperitone**	28.39	0.14
**Spathulenol**	42.09	0.10
**Total content**	**-**	93.26

The MIC and MBC values of the EOs against tested bacterial strains are shown in Table 3. According to the results, both EOs had the lowest MIC and MBC values (0.12 and 0.25 mg mL^-1^, respectively) against *S. aureus*. The highest MIC and MBC values *of D. moldavica* EO were observed against *E. coli* and *S.typhimorium* with the same values (MIC: 2.00 mg mL^-1^, MBC: 4.00 mg mL^-1^). *E. coli *was the most resistant bacteria to *M. officinalis *EO with similar MIC and MBC values (2.00 mg mL^-1^). 

**Table 3.** Antibacterial activity of *M. officinalis* *and D. moldavica* Eos determined by agar disk diffusion assay and micro-well dilution assay

**Bacteria**	**MIC **(**mg mL**^-1^**)**		**MBC **(**mg mL**^-1^**)**		**Diameter of inhibition zone (mm)**					
	**MEO**	**DEO**	**MEO**	**DEO**	**MEO (mg mL** ^-1^ **)**		**DEO (mg mL** ^-1^ **)**		**Vancomycin**	**Ampicillin**
					**10**	**15**	**10**	**15**		
***E. coli***	2.00	2.00	2.00	4.00	9.74	11.17	8.21	9.25	NG	18.20
***S. typhimurium***	1.00	2.00	2.00	4.00	8.87	10.12	8.55	9.27	NG	18.80
***S. aureus***	0.12	0.12	0.25	0.25	10.60	15.00	9.53	12.15	NG	NG
***L. monocytogenes***	1.00	2.00	1.00	4.00	10.45	15.10	9.11	11.21	26.30	30.20

MEO: *M. officinalis* Essential oil; DEO: *D. moldavica* Essential oil; NG: No growth.

**Antioxidant properties. **Results of *in vitro* antioxidant properties of *M. officinalis* and *D. moldavica* EOs by DPPH assay are presented in Table 4. Similar concentrations of ascorbic acid and BHT were used to compare the antioxidant potency. The scavenging potency of the oils was dose dependent and increased with the increment of EOs concentration.

Table 4 represents the results of ABTS test of *M. officinalis* and *D. moldavica* EOs. The same concentration of ascorbic acid and BHT were used for comparison. Both EOs showed a strong activity in maintenance of β-carotene molecules which was higher than that of ascorbic acid.

Results of ABTS assay of *M. officinalis* and *D. moldavica* Eos and ascorbic acid and BHT are presented in Table 4. The ABTS radical scavenging of the EOs was dose dependent and increased with the increment of the EOs concentration.

**Table 4.** Antioxidant activities of *M. officinalis *and* D. moldavica*

**Sample**	**Test (%)**	**Concentration ** **(mg mL** ^-1^ **)**			
		**1**	**2.5**	**5**	**10**
***M*** ***.*** *** officinalis***	**DPPH**	21.70 ± 2.17	34.80 ± 1.40	49.36 ± 0.00	71.43 ± 2.81
	**BCBT**	12.20 ± 2.18	23.50 ± 1.75	38.85 ± 1.21	51.25 ± 1.15
	**ABST**	15.05 ± 0.39	29.76 ± 0.86	47.28 ± 2.22	65.53 ± 1.45
***D*** ***.*** *** moldavica***	**DPPH**	11.40 ± 2.25	21.50 ± 3.10	28.79 ± 1.10	36.44 ± 2.21
	**BCBT**	12.14 ± 4.36	22.93 ± 3.25	36.47 ± 2.13	49.75 ± 4.01
	**ABST**	14.19 ± 1.19	27.55 ± 3.25	43.28 ± 3.78	60.81 ± 3.50
**Ascorbic acid**	**DPPH**	98.40 ± 0.09	98.75 ± 0.00	99.16 ± 0.01	99.65 ± 0.09
	**BCBT**	9.52 ± 2.20	12.33 ± 3.11	17.94 ± 2.45	27.51 ± 3.58
	**ABST**	98.4 ± 0.09	98.75 ± 0.00	99.16 ± 0.01	99.65 ± 0.09
**BHT**	**DPPH**	98.19 ± 0.19	98.68 ± 0.09	99.37 ± 0.09	99.72 ± 0.19
	**BCBT**	82.34 ± 0.74	88.53 ± 0.43	95.83 ± 0.52	99.99 ± 0.00
	**ABST**	98.19 ± 0.19	98.68 ± 0.09	99.37 ± 0.09	99.72 ± 0.19

DPPH: 2,2- diphenyl-picrylhydrazyl test; ABTS: 2,2'-azino-bis (3-ethyl benzo thiazoline-6-sulphonic acid test; BCBT: β-carotene bleaching test; BHT: Butylated hydroxytoluene

## Discussion

The chemical composition of the EO extracted from *D. moldavica* harvested in Urmia, Iran, was dominated by geranial, neral, geraniol and geranyl acetate, respectively. These chemical compositions were similar to the chemical composition of the oils from Egypt^18^^,^^19^ and Hungary.^20^ The results also showed that *M. officinalis* EO was characterized by the presence of four dominating components: Citronellal, Thymol, Citral and β-caryophyllene, respectively. In a study in which EOs obtained from aerial parts of *M. officinalis* grown in Turkey were analyzed by GC-MS, citronellal (36.62 to 43.78%), citral (10.10 to 17.43%), thymol (0.40 to 11.94%), and β-caryophyllene (5.91 to 7.27%) were recorded as the major components,^21^ which they were completely consistent with the results of the present study. However, the variations in the essential oil composition may be due to the region’s climate, plant species, distillation conditions and maturity stage and some other factors.^22^

The advantage of essential oils and their biological properties have driven recent researches to the direct evaluation of antioxidant and antimicrobial activity of plant EOs *in vitro *and also in food models.^23^^,^^24^ Disk diffusion agar and micro-dilution tests are known assays to identify antibacterial properties of the EOs.^25^ The results of this study revealed that gram-positive bacteria were more sensitive than gram-negative bacteria to the tested EOs. This finding was completely consistent with the literature data.^25^^-^^29^ Divalent cations and polysaccharide part of lipopolysaccharides in the outer cell membrane of gram-negative bacteria obtains hydrophilic qualities that impede the contact of the hydrophobic constituents (such as EOs) with the bacterial cell, resulting in a higher resistance of gram-negative bacteria to the antibacterial properties of the EOs.^13^^,^^25^^,^^27^^,^^29^^,^^30^

According to the disk diffusion agar assay and micro-dilution method, it can be concluded that *S. aureus* had the highest sensitivity to *M. officinalis *and* D. moldavica* EOs. Several studies have shown that *S. aureus* is the most sensitive bacteria against essential oils.^31^ However, there have been few studies regarding antibacterial effects of *M. officinalis *and *D. moldavica *EOs.^32^ In a study carried out on several gram positive and gram negative bacteria, *E.* *coli *and the multi-resistant strain of *Shigella sonei *have shown the highest sensitivity to the antibacterial effect of *M. officinalis *EO. In another study,* M. officinalis *EO revealed strong antimicrobial activity against *E. coli* and *S. enterica.*^33^ This variation can be due to the differences in major and/or minor components of the EOs. Various factors can affect EOs chemical composition such as climate, seasonal and geographic conditions.^22^^,^^31^

 Nowadays consumers prefer to use EOs and plant extracts instead of chemical preservatives in order to prevent microbial growth and oil oxidation.^34^ Using DPPH and ABST assays for evaluating antioxidant activity of the EOs in the present study revealed that both EOs were weaker than ascorbic acid and BHT. Although, BCBT indicated a higher activity of *M. officinalis* and *D. moldavica *EO than ascorbic acid. These results were in agreement with those reported in former studies.^35^^-^^37^ There are some other studies indicating the both of these EOs can be used as antioxidant as well.^37^^-^^40^ There is a direct correlation between the free radical scavenging and antioxidant activity of the essential oils and the concentration. Also, a linear trend has been observed between polyphenolic concentration and radical scavenging activity of different plants.^22^ The antioxidant potency of both EOs may be due to the presence of different bioactive EOs compounds which are identified by GC-MS analysis such as flavonoids, geraniol, thymol, geranial, citronellal and citral.^41^

In conclusions, the present study revealed a significant antimicrobial effect of *M. officinalis* and *D. moldavica *against *S. typhimorium, E. coli, L. monocytogenes *and* S. aureus*. Although *M. officinalis* EO showed better antimicrobial properties than *D. moldavica* EO. On the other hand, a remarkable potency was identified in antioxidant activity of both EOs using different assays, therefore, they can be used as natural remedies in infectious diseases as well as natural preservatives in food industries.
